# Tackling the global health threat of arboviruses: An appraisal of the three holistic approaches to health

**DOI:** 10.34172/hpp.2021.48

**Published:** 2021-12-19

**Authors:** Yusuf Amuda Tajudeen, Iyiola Olatunji Oladunjoye, Mutiat Oluwakemi Mustapha, Sheriff Taye Mustapha, Nimat Toyosi Ajide-Bamigboye

**Affiliations:** Department of Microbiology, Faculty of Life Sciences, University of Ilorin, Nigeria

**Keywords:** Concept of health, Arboviruses, Public health practice, Ecology, Medicine, Veterinary medicine, Holistic health

## Abstract

**Background:** The rapid circulation of arboviruses in the human population has been linked with changes in climatic, environmental, and socio-economic conditions. These changes are known to alter the transmission cycles of arboviruses involving the anthropophilic vectors and thus facilitate an extensive geographical distribution of medically important arboviral diseases, thereby posing a significant health threat. Using our current understanding and assessment of relevant literature, this review aimed to understand the underlying factors promoting the spread of arboviruses and how the three most renowned interdisciplinary and holistic approaches to health such as One Health, Eco-Health, and Planetary Health can be a panacea for control of arboviruses.

**Methods:** A comprehensive structured search of relevant databases such as Medline, PubMed, WHO, Scopus, Science Direct, DOAJ, AJOL, and Google Scholar was conducted to identify recent articles on arboviruses and holistic approaches to health using the keywords including "arboviral diseases", "arbovirus vectors", "arboviral infections", "epidemiology of arboviruses", "holistic approaches", "One Health", "Eco-Health", and "Planetary Health".

**Results:** Changes in climatic factors like temperature, humidity, and precipitation support the growth, breeding, and fecundity of arthropod vectors transmitting the arboviral diseases. Increased human migration and urbanization due to socio-economic factors play an important role in population increase leading to the rapid geographical distribution of arthropod vectors and transmission of arboviral diseases. Medical factors like misdiagnosis and misclassification also contribute to the spread of arboviruses.

**Conclusion:** This review highlights two important findings: First, climatic, environmental, socio-economic, and medical factors influence the constant distributions of arthropod vectors. Second, either of the three holistic approaches or a combination of any two can be adopted on arboviral disease control. Our findings underline the need for holistic approaches as the best strategy to mitigating and controlling the emerging and reemerging arboviruses.

## Introduction

The emergence and re-emergence of arthropod-borne viruses otherwise known as arboviruses is a public health threat across the globe causing an increased infection to humans and animals, thus, resulting in a large socio-economic burden. As reported by the World Health Organization (WHO), vector-borne diseases (VBDs) including arboviruses account for ~17% of infectious diseases and about 700 000 deaths per annum worldwide.^1^ For transmission to occur, arboviruses require arthropod vectors such as mosquitoes, sandflies, and ticks to allow for their replication before transmission to their vertebrate host – mostly humans and animals.^2^ Arboviruses undergo series of cycles such as sylvatic or enzootic cycle where disease transmission occurs between wild animals serving as the reservoir host and the arthropod vectors, this has the tendency of increased viral amplification in the host with humans as the dead-end host through spillover.^2,3^ Epizootic cycle involves the transmission of diseases between domestic animals and arthropod vectors and has the potential of causing an epidemic in the animal population; humans get infected during contact with infected animals.^2,3^ Arboviruses also undergo an urban cycle where humans are the amplifying host as well as the source of infections causing an epidemic in the urban area.^3^ Infections posed by arboviruses to humans and animals are concentrated in four families which are *Togaviridae, Flaviviridae, Bunyaviridae,*and *Reoviridae.*^4^ However, increased arboviral infections are caused as a result of increased geographical distribution and abundance of arthropod vectors especially the species of *Aedes* (*Aedes aegypti*and* Aedes albopictus*) and *Culex*(*Culex tarsalis, Culex quinquefasciatus, Culex pipiens*) mosquitoes known to transmit medically-important arboviral diseases such as West Nile viruses (WNVs), yellow fever viruses (YFVs), Chikungunya viruses (CHIKVs), and dengue viruses (DENVs).^5^ Other prevalent vectors such as sandflies, ticks, and *Culicoides* are associated with the transmission of arboviruses.^5^ Consequently, the spread of arboviruses is on the rise in an era pronounced as Anthropocene (a recently proposed epoch in the geological time scale and the era of humans), and this is closely tied to concurring factors including migration, urbanization, climate change, and others affecting the viral agent, the human host, and the environment.^6^ In a bid to safeguard human health against public health threats i.e. emerging and reemerging infectious diseases, several holistic approaches like One Health, Eco-Health, and Planetary Health have been developed and adopted by the global health communities. This article, therefore, highlights arboviruses as a global health threat, explicates some important arboviral diseases and factors facilitating their spread, and finally appraised the three relevant holistic approaches as a possible way forward.

## Materials and Methods

We conducted a narrative review in search of relevant articles published in English on arboviruses and holistic approaches from 1990 to 2021. In our search, we considered a number of databases and search engines including Medline, PubMed, WHO, Scopus, Science Direct, DOAJ, AJOL, and Google Scholar to identify relevant articles using the following keywords and search terms; “arboviral diseases”, “arbovirus vectors”, “arboviral infections”, “epidemiology of arboviruses”, “holistic approaches”, “One Health”, “Eco-Health”, and “Planetary Health”. Relevant articles including epidemiological and clinical studies of arboviruses were critically accessed for initial eligibility in line with our study. Studies that were evaluated and appraised depict the association and relationship between arboviruses and one or more of the holistic approaches to health including One Health, Planetary Health and EcoHealth.

## Important arboviral diseases

### West Nile fever

WNV is a mosquito-borne flavivirus in the Japanese encephalitis serogroup and belongs to the *Flaviviridae* family.^2^ Since the first case of the virus was reported in Uganda in 1937, it has continued to spread across different parts of the world including Israel, Russia, the United State of America, and Romania where the largest epidemic has occurred.^7,8^ Through an enzootic transmission cycle, the virus is transmitted between *Culex* mosquitoes – the principal vectors of the virus – and birds – the reservoir hosts, and humans are just accidental hosts when spillovers occur during an outbreak in the animals’ population.^2^ In the urban transmission cycle, human infections can also occur through the bite of infected *Culex*mosquitoes transmitting the virus.^9^ Changes in climate factors (temperature, precipitation, humidity) and other human factors like migration, land-use change, and urbanization played an important role in the dissemination of WNV which have been documented in various studies.^10-14^ Temperature as an important climatic factor is closely linked with the distribution of *Culex*mosquitoes which are sensitive to even a little change in ambient temperature. In their study, Kilpatrick and colleagues reported that increased temperatures accelerate the frequency of mosquito bites and viral replication rate of WNV in vectors, which in turn lead to a high rate of WNV infections.^12^ Migration of people between countries as a result of trade and travel has led to the introduction of important mosquito vectors of WNV including *C. pipiens* and *C. quinquefasciatus* between countries.^14^ Prevalence of *C. pipiens* and *C. tarsalis,* the two most important WNV mosquito vectors in eastern and western North America, are associated with increased anthropogenic land-use change, urbanization, and intensified agricultural practices.^15,16^ To date, the human-to-human transmission via contact of the virus has neither been reported nor documented and a human vaccine against the virus is yet to be developed. Cases of arbovirus infection being misdiagnosed and treated like other diseases such as malaria, hepatitis, measles, and coronavirus diseases 2019 (COVID-19) have been reported.^17-22^ In a study by Schiuma and colleagues, WNV infection being misdiagnosed for COVID-19 in an Egyptian woman has been reported.^22^

### Zika 

Zika virus (ZIKV) is another mosquito-borne flavivirus that belongs to the family *Flaviviridae*.^23^ The virus was first discovered from the serum of sentinel monkeys in 1947 and is named after a forest – Zika – in Uganda where the monkey was found.^24^ The following year, the virus was also discovered from *Aedes*mosquitoes in the same forest and thus, it can be concluded that the virus has its origin in Africa. The virus is transmitted by *Aedes* mosquito vector species including *Ae. albopictus, Ae. hensilli, Ae. poliniensis*, and *Ae. aegypti*—the principal vector of WNV associated with urban transmission.^25^ The first human case of infection was discovered in a Nigerian lady and since then, it has continued to spread at an alarming rate to different regions of African countries thereby causing an epidemic.^26,27^ However, the epidemic of the virus has also been reported outside the African region such as USA, Asia, and French Polynesia where it poses a significant threat to human health.^28,29^ Increased temperature favors the activities (fecundity, survival, biting, and competence) of *Ae. aegypti* and this has been associated with the emergence of ZIKV in Americas during the summer period in both tropical and subtropical regions of the country.^30^ Anthropogenic human activities like urbanization and land-use change that could result in decreased land cover have been linked with increased *Ae. aegypti*activities and transmission of ZIKV infections.^30^ Consequently, migration of infected people and trading of old used tires between countries are associated with increased ZIKV infections.^31^ Medical factors such as misdiagnosis also occur in ZIKV as reported by Oidtman and colleagues in a study where ZIKV was misdiagnosed for CHIKV or Dengue across the Americas and has thus led to the underestimation of ZIKV epidemic that occurred in 2015-2017.^32^

### Chikungunya

CHIKV is an Alphavirus that belongs to the *Togaviridae* family.^33^ The virus which has its origin in the eastern half of Africa is widely transmitted throughout the world by *Ae. aegypti*and* A*e.* albopictus* thereby causing epidemics.^33^ However, studies have shown that the virus circulates rapidly in the enzootic cycle among the non-human primates through *Aedes* vectors while humans may be an accidental host through the bite of *Ae. albopictus*, the main vector implicated in the spread to humans.^34^ The virus also undergoes sylvatic transmission cycle between arboreal mosquitoes and wild primates, and this has been reported in Africa.^2^In 1952, the epidemic of the virus occurred in Tanzania and was reported as the first diagnosed case of CHIKV.^35^ Since the first epidemic, several other outbreaks have been reported in Africa and South East Asia where transmission of the virus occurred through the urban cycle involving the mosquito vectors and the human host.^36,37^ An increased epidemic of CHIKV has been associated with its genetic mutation facilitating multiplication within the midgut of *Aedes* mosquito vectors.^37^ Climatic factors are also associated with the epidemics of CHIKV, as an increased temperature has been linked with its first outbreaks in Italy.^38,39^ Consequently, heavy rainfall from increased precipitation was linked with increased transmission of CHIKV in France in 2014 as a result of an abundance of *Ae. albopictus.*^40^ Cases of misdiagnosis of CHIKV for other arboviral diseases occur and have been discussed in several pieces of scientific literature.^41,42^

### Japanese encephalitis

Japanese encephalitis virus (JEV) is a Flavivirus of the *Flaviviridae* family widely distributed across the world and causing a public health threat.^43^ The virus is transmitted by *Culex tritaeniorhynchus*and* C. pipiens*. In the enzootic cycle, the virus is transmitted between *Culex* mosquitoes and vertebrate species like aquatic birds and pigs, while humans and equids are the dead-end hosts.^44^ Humans become infected through the bite of infected *C. tritaeniorhynchus* when the virus is amplified in the domestic cycle by pigs due to their proximity to human habitat as elucidated in a study by Yun and colleagues.^45^ Histologically, the first confirmed case of the virus was reported in Japan in 1924 and since then, cases have been confirmed in other countries such as India, the Philippines, Korea, and other parts of the world but particularly the Asian countries.^43,46^ However, the virus is endemic in some parts of Asia as a result of increased population and intensified rice farming cultivation facilitating the spread of *Culex* mosquito vectors.^47^ In a study by Preziuso and colleagues, JEV has been detected in the bone marrow of migratory wild birds in Italy suspected to be due to the migration of the bird from Asia – where the virus is endemic – to Europe.^48^ In their study, Hsu and colleagues established a positive correlation of climatic factors (temperature and precipitation) with the increased spread of JEV.^49^ A decrease in temperature as a result of lower rainfall has led to a decrease in *C. tritaeniorhynchus*mosquito in 1980-1981 in a study in China.^49^ Consequently, medical factors like misdiagnosis also occur in JEV as reported in various studies.^50^

### Yellow fever

YFV is a mosquito-borne flavivirus propagated mainly by the *Aedes* species of mosquitoes, and sometimes the *Haemagogus* sp. and the *Sabethes*sp.*—*found mostly in forested areas.^51^ Yellow fever is endemic to 47 countries in Africa, and Central and South America.^52^ Angola including the Democratic Republic of the Congo (DRC) experienced large outbreaks of yellow fever in 2015 and 2016 while Brazil and Nigeria followed in 2017 and 2018 respectively.^52^ Higher cases have been recorded in Brazil due to the expansion of the epizootic endemic areas to places close to Rio de Janeiro and São Paulo – two of the major cities in the country.^53^ Yellow fever outbreaks continue to occur in Africa, affecting both rural and urban populations, and in South America. Infection by the *Haemagogus* sp.and the *Sabethessp* affect mostly male humans and it is suspected to have started with men who entered forested areas for work or recreation as associated with recent outbreaks. *Ae. aegypti,* the species causing infection in the urban communities of tropical and subtropical South America, has not been documented in recent outbreaks,^54^ the last urban outbreak in Brazil being in 1942. The virus genomes recognized from the South America outbreak in December 2016 further proved that infection was due to forest YFV spilling over into human populations, and this caused more than 2000 human cases and more than 700 deaths in Brazil alone within 2 years, graduating into the largest epidemic in Brazil for decades.^55^ Climatic and other environmental factors have been associated with the spread of YFV and this has appeared in various scientific studies.^56-58^ Misdiagnosis of YFV has also been reported.^58^

### Dengue fever

Dengue fever (DF) is the fastest spreading mosquito-borne viral infection and is caused by the dengue virus (DENV), a flavivirus of the *Flaviviridae* family.^59^ DF is considered an important health challenge in 128 countries^60-63^ in the tropic and subtropic nations^64^ with an estimate of 3.97 billion people residing in dengue-endemic regions^65^ and about 400 million infections recorded per year.^66-69^ The Indian subcontinent has been identified as the center of DF^70^ and having a highly underestimated number of cases.^71^ The first reported case of a dengue-like illness in India was in 1780, but there was no proven case until the 1963 epidemic.^72^ Countries in Africa such as Angola, Benin, Burkina Faso, Cape Verde, Comoros, Egypt, Ivory Coast, Kenya, Nigeria, Somalia, Tanzania, and Zanzibar have suffered dengue epidemics since 2010^73,74^; with sporadic DENV outbreaks having been reported in Nigeria since 1960.^75^ The virus is widely distributed, not only in the urban environment but also in the forest areas of the tropical, subtropical and temperate regions of the world through two important species of the *Aedes* mosquito vectors (*Ae. aegypti*and* Ae. albopictus*).^76^ Humans are the reservoir and amplifying host of the virus and usually got infected through the bites of *Aedes* mosquitoes.^76^ Various climatic factors such as temperature, precipitation, as well as other factors like urbanization and migration have been linked with the distribution of *Aedes* mosquitoes which in turn facilitate the spread of dengue.^77-79^ Misdiagnosis of the virus has also been reported.^80^

## Factors associated with the spread of arboviral diseases

### Error in classification and diagnosis

In Sudan and other parts of Africa, misclassification and misdiagnosis are major challenges associated with the spread of arboviruses. This is due to the likeness of symptoms and signs between arboviral diseases and other local infectious diseases, e.g. malaria, hepatitis, measles, etc.^17-21^ Also included are the unavailability of specified health policies for the prevention and control of these diseases,^81^ almost non-existent diagnostic power,^20,82,83^ and the fact that health funds are mainly diverted to preventing and treating malaria in malaria-endemic countries and territories.^84^

### Climatic factors

Climatic factors such as temperature, humidity, and precipitation encourage the growth, survival, and dispersal of JEV mosquito vectors.^10,85,86^ In Asian countries such as China, Japanese encephalitis has an established pattern that varies with the season in humans. There is always an increase in the number of cases from June which goes on to and heightens in August, continues till September when it starts to decrease.^86,87^ In India, also, there is a recorded relationship between temperature and mosquito density in areas that are endemic to JEV, showing optimal temperatures for the vectors to lie between 22.8-34.5^o^C.^88,89^ The higher the temperature, the higher the development and survival rate of mosquito larvae in the cold, and the shorter the incubation period for replication in mosquitoes, and also the time between blood meals. Hence, the reduction in time for transmission from the mosquito vector to animal and human hosts.^90^ Therefore, higher temperatures give way to the rapid increase in mosquito populations to reach large population sizes, which then facilitates JEV transmission.^91,92^

However, the correlations with precipitation, density, and temperature are not always linear and this can be seen in the WNV.^10,93^ Examples are laboratory studies that have shown that the infection and transmission rates in WNV increase with temperature in some mosquito types but not in others,^94^ and that some strains of some viruses are transmitted better in higher temperatures than others.^95^ Also, the precipitation effect of the epidemiology of WNV differs with regions and is likely dependable on the species and lineage of the vector.^93^

As for the Rift Valley fever virus, epidemics usually break out after heavy rainfall and flooding leading to the creation of temporary mosquito breeding pools that allow for the breeding of mosquitoes into large numbers.^10,96-101^ Still, some previous outbreaks in some parts of Africa were not associated with heavy rainfall, neither do they occur during droughts.^102,103^ As with all and other mosquito-borne viruses, temperature also plays a very great role in the ecology of Rift Valley fever virus.^101,104,105^ Temperatures from 17-35°C allow the increase of mosquito populations, enabling them to reach large population sizes for longer periods,^76,106^ facilitating replication and transmission in *Culex* spp^107^ and *Aedes* spp^108^ mosquitoes.

### Migration

According to the United Nations prediction, the global population tends to increase from its currently >7 billion population to about 9.6 billion by 2050.^109^ The major consequence of this population increase is the ability to facilitate the increased transmission of vectors and the viruses they are transmitting. As the global population increases, a large number of people tends to change their location by way of migration thus facilitating the spread of viruses directly from person-person or through vectors. Consequently, while most arboviral diseases are endemic to the areas where they are recorded, others found their way there via several means ranging from the slave trade to migration of birds and livestock, and mobility of humans from the endemic countries to countries where the viruses are novel: YFV and DENV to the Americas, CHIKV to the New World and Polynesia; yet others reemerged or were reintroduced after previously being eradicated.^110^

### Abundance in propagative vectors

The availability of different species and types of vectors able to transmit arboviruses plays an important role in their rapid transmission and difficult eradication, prevention, control, and treatment. 300 types of mosquitoes (mainly *Aedes* spp. and *Culex* spp.),^111^ 116 types of ticks, 25 species of midges* (*majorly*Culicoides* spp. and *Lasiohelea*spp.), bedbugs, mites, stinkbugs,sandflies, gadfly, lice, blackflies, etc,^112^ all aid the propagation of arboviruses.^2,111,113^ Also, some arboviruses known to be transmitted by a specific type of vector are transmitted by others. For example, WNV is known to be mosquito-borne, but its propagation and transmission via ticks and other arthropods have been recorded.^2,111,114^ Also, JEV can be transmitted by *Culex*, *Anopheles*, other mosquito species, midges, sandflies, and ticks.^115^

### Urbanization

Overpopulation of urban areas, migration of humans, the establishment of settlements, human activities, etc. all contribute to the emergence and reemergence of most arboviruses. As vectors need breeding grounds – mostly standing water – as well as hosts – humans and animals – these requirements are satisfied where there is human inhabitation, and even better if the population is high. Examples of arboviruses implicated in this are DENV^116^ and WNV.^11,117-119^

### Changes in land use

This has been linked with loss of vectors’ and host’s habitats which affect their distribution patterns including their behavioral changes, as vectors tend to search for new breeding sites and habitats. There is a relationship between the spread of JEV and WNV and rice farming, pig farming, poultry farming, etc.^11,120-122^

## Holistic approaches as a panacea: Planetary Health, One Health, and EcoHealth

Newly emerging and re-emerging arboviral diseases such as yellow fever, Zika, and Chikungunya continue to pose a public health threat to the human populations owing largely to their distributions which are greatly affected by environmental, societal, and human-associated factors.^76,123-125^ Over the years, several strategies including biological, chemical, and physical control^126^ have been adopted on VBDs but most have failed while the prevalence of arboviruses persists, this is evident from the recent epidemic outbreak of ZIKV throughout the United States in 2016,^127^ and the Jaipur city of India in 2018.^128^ However, coping with emerging and re-emerging arboviral disease epidemics requires the adoption of a holistic approach that works with the vector-host-environment interface, involving experts from interrelated fields to perform transdisciplinary and evidence-based research aimed at promoting VBDs prevention, surveillance, management, and control. It is pertinent to note that several holistic approaches exist on health security against public health threat, and the most relevant ones are Planetary Health, One Health, and EcoHealth.^129^ Of these three, the first is a new concept and appears to encapsulate the other two. The appraisal of the holistic approaches to health towards improving arboviral disease control have been summarized in Table 1.

**Table 1:** Summary on the appraisal of holistic approaches to health towards improving arboviral disease control

**Holistic approaches**	**Recommendations**
Planetary Health	Interdisciplinary coordination on entomological surveillance and vector control monitoring programme should be activated.
	Integration of Reference Laboratory across the country involving expertise to collaborate on diagnostic procedures.
	Introduction of National Policy and Regional Framework aimed at integrating vector control programme and surveillance into national healthcare system across the country.
	Availability of funds from donors’ organization, private institutions, and government to actualize the programme.
EcoHealth	Elimination of breeding site of vectors that transmit arboviral diseases.
	Routine environmental sanitation exercises that promote clean environment & National Vector Control programme
	Community advocacy on the use of insecticide-treated nets.
	Adoption of latest molecular technology such as CRISPR-Cas9 to reduce vectors population.
	Training of physicians on advanced methods of VBD control.
	Provision of antiviral drugs, vaccine, and diagnostic equipment at primary, secondary, and tertiary healthcare facilities respectively
One Health	Prioritization of One Health based research by researchers on arboviral diseases especially on factors determining the transmission and infection of VBD, integrated surveillance system, development of effective control therapy including antiviral agents and vaccine.
	Assessment and evaluation of existing interventions, policies, and frameworks for arboviral disease control.
	Interdisciplinary coordinated VBDs surveillance and monitoring of local vectors of arboviral diseases should be conducted.

## Planetary Health

Since global climate changes caused by anthropogenic activities are an important driver of VBDs,^130,131^ the adoption of a planetary health approach could be the best strategy that can make us see the connection between our health and the environment, thereby helping to cope with the emergence of arboviruses. The Rockefeller Foundation-*Lancet*Commission clearly defined planetary health in their article published in 2015 as: “the achievement of the highest attainable standard of health, wellbeing, and equity worldwide through judicious attention to the human systems – political, economic, and social – that shape the future of humanity and the Earth’s natural systems that define the safe environmental limits within which humanity can flourish”.^132^ From this definition, it is apparent that the planetary health approach aims to mitigate and address the diverse effects of environmental health threats faced by humans to achieve optimal health and well-being.^132^ Planetary Health is a novel concept developed by the Rockefeller Foundation in 2015 to understand the effects of global environmental changes on earth’s natural systems, and how these effects affect human health and well-being at different levels,^133^ this concept extends to understand the influence of global climate change and temperature on the distribution of emerging infectious diseases outbreaks to ensure a sustainable environment for future generations to thrive mentally and physically.^60^

## Planetary Health as a panacea to arboviral diseases

The Planetary Health concept can be adopted by the global communities in response to the emerging and re-emerging VBDs in the following ways:

Well-coordinated interdisciplinary entomological surveillance and vector control monitoring should be implemented for early detection and prompt response to emerging arboviral diseases.^61,62^ Adopting a Planetary Health approach will be of great benefit in tackling the emergence of VBDs particularly in resource-limited settings including the developing African countries as it allows intersectoral collaboration leading to cost-resource sharing amongst translational fields within the concerned ministries.

There should be an integrated national reference laboratory across each country involving expertise collaboration to share ideas and step-by-step procedures on the diagnosis of arboviral diseases, interpretation of results, and data sharing.^63^ This will ensure quality data are presented for adequate national treatment guidelines.

National policy and regional frameworks aimed at integrating vector control and surveillance in the health systems across the countries should be implemented.^134^ This would enhance preparedness and response to outbreaks of VBDs.

Planetary Health as a novel approach requires huge funding for implementation,^38,135^ and thereby requires support from the government and policymakers through partnership and engagement with Planetary Health stakeholders to facilitate the management, control, and response to arboviral diseases.

## One Health

One Health has been described as a concept that aims to understand the interconnections that exist between humans, animals, plants, and their shared environments to optimize their health through interdisciplinary and intersectoral collaborations amongst expertise at the global, regional, national, and local level.^136^ This concept, therefore, tends to safeguard the health of humans, animals, and the environment by addressing the public health threats occurring at their shared interface.^137^ The One Health concept is one of the oldest of the three concepts and the history dates back to 1980s when scientists underline the need to tackle and protect the health of humans and animals from global health threats of infectious diseases such as VBDs and zoonoses.^138^ In the fight against VBDs, the One Health concept can be adopted by the global communities as a mitigating strategy for arboviruses.

## One Health as a Panacea to arboviral diseases

The rise and spread of arboviral diseases threaten the global communities including the developed, developing, and underdeveloped countries.^36,76,106,123^ However, adopting the One Health concept can be the best control and mitigation strategy for the emerging arboviral diseases in the following ways:

An interdisciplinary coordinated VBDs surveillance and monitoring of local vectors of arboviruses should be conducted; this is to understand the pattern of spread and transmission of arboviral diseases.

Since there are challenges with the diagnosis of arboviral diseases such as misdiagnosis and indistinguishable infection symptoms due to the co-circulation of arboviruses, there should be an availability of a national reference laboratory for arboviral diseases where differential diagnosis and specific serological tests for seroprevalence studies would be performed.^76^ This should involve the collaboration of relevant expertise to share ideas, knowledge, and skills for effective confirmation of arboviral diseases on time.

One Health-based research on arboviral diseases should be prioritized amongst researchers particularly on factors determining the transmission and infections of VBDs, surveillance systems, development of effective control therapy including antiviral drugs and vaccines.^76^

There is a need for assessment and evaluation of existing interventions, policies, and frameworks for arboviruses control. This is to ensure appropriate and up-to-date contingency plans for arboviral disease epidemics.

## EcoHealth

EcoHealth is an interdisciplinary systems-based concept aimed at promoting the health of humans, animals, and ecosystems but focusing more on ecological and socio-economic stability.^139^ Waltner-Toews,^140^ in his review, suggested that the primary aim of EcoHealth is to ensure the sustainable health of humans and animals through a flourishing ecosystem. This old concept has a more focus on the effect of biodiversity conditions on emerging infectious diseases with a view that zoonotic pathogens and VBDs are of wildlife origin.^129,139,140^ It, therefore, seeks to reduce and address the health crises in humans, animals, and the environment caused as a result of ecological disruptions, particularly the loss of biodiversity due to anthropogenic activities. Since arboviral diseases have a biodiversity origin, the adoption of an EcoHealth concept could be the best strategy for control.

## EcoHealth as a Panacea to arboviral diseases

Due to the prevalence of arboviral diseases, it is essential to prevent the transmission of pathogens by vectors, and this can be achieved through the adoption of the EcoHealth concept in the following ways:

An interdisciplinary and intersectoral collaboration amongst expertise and relevant ministries aimed at environmental management should be performed.^76^ This includes the elimination of the natural breeding site of vectors, adoption of good environmental sanitation practices that reduce human-vector contact, increased advocacy to people on the use of insecticide-treated bed nets.^76,141^

Existing policies and interventions on vector control should be evaluated and screened while new models and policies approach such as the use of genetic engineering techniques like CRISPR-Cas9 that allows targeted modification and transformation of vectors’ genes could be the way to reduce vector populations.^142^

Advocacy to the population and training of physicians on VBDs control should be carried out at the local and national levels.^36^ This will enable individuals to be enlightened on the best control strategies for VBDs, at the same time physicians should be equipped with the latest skills and methodologies of arboviral disease control.^36^

Vector control programs and VBDs surveillance supported by the government should be carried out regularly, while antiviral drugs, vaccines, and diagnostic tools should be made available in the national healthcare sector. This will increase the national health capacity response to arboviral diseases control (Table 1).

## Conclusion

The geographical distribution of arboviruses being facilitated by changes in climatic, environmental, and socio-economic conditions is a public health threat across the globe. To safeguard the health of humans and that of animals from the risk of infections posed by arboviruses, several holistic approaches have been developed. However, the three most relevant are One Health, Eco-Health, and Planetary Health – these approaches have a lot in common because of their sole aim in protecting the health of humans, animals, and their shared environment from any known public health threat. However, there are also some differences in their core values which may affect their method of application to solving a public health threat. In this review, we have highlighted arboviruses as a global public health threat and explicated some factors facilitating their geographical distribution. Consequently, considering the interconnection that exists between the health of humans, animals, and their shared environment, we appraised the approaches as a way forward to mitigate the public health threat of arboviruses. This review was consciously prepared without being sentimental or prioritizing an approach over the other as we finally conclude that either of these approaches can be adopted on arboviral diseases control or a combination of any two.

## Acknowledgements

The authors appreciate Dr. Habiba Atta and the reviewers for their insightful comments in improving the quality of this manuscript. YAT and IOO also extend their appreciation to the Africa Community of Planetary Partners for Health and Environment (ACOPPHE)’s Mentoring Research Network whom they have gained a lot of inspiration and knowledge from, through experiential learning and meaningful academic conversations in the fields of One Health, EcoHealth, and Planetary Health.

## Funding

None.

## Competing Interests

None.

## Ethical Approval

Not applicable.

## Authors’ contributions

The concept for this review was developed by YAT and IOO. YAT, IOO, MOM and STM developed the draft and prepared the manuscript. YAT, IOO and NTA assisted with data collection, article interpretation, and language edits. All the authors have read and agreed to the final manuscript.
